# Tetra­aqua­bis(4,4′-bipyridine)zinc(II) bis­(*trans*-4-hydroxy­cinnamate)

**DOI:** 10.1107/S1600536809023204

**Published:** 2009-06-20

**Authors:** Ling Chen

**Affiliations:** a College of Chemical Engineering and Pharmacy, Jinhua College of Profession and Technology, Jinhua, Zhejiang 321017, People’s Republic of China

## Abstract

The title complex, [Zn(C_10_H_8_N_2_)_2_(H_2_O)_4_](C_9_H_7_O_3_)_2_, was obtained by the hydro­thermal reaction of zinc sulfate with mixed 4-hydroxy­lcinnamic acid (H_2_ *L*) and 4,4′-bipyridine (4,4′-bipy) ligands. The complex consists of a centrosymmetric [Zn(4,4′-bipy)_2_(H_2_O)_4_]^2+^ cation with the metal centre in a distorted ZnN_2_O_4_ coordination, and of two H*L* ^−^ anions. Extensive O—H⋯O and O—H⋯N hydrogen-bonding inter­actions between the constituents lead to the formation of a three-dimensional network.

## Related literature

The main strategy used in the design and synthesis of novel coordination architectures is the building-block approach, see: Han *et al.* (2005 ▶); Wen *et al.* (2005 ▶); Yaghi *et al.* (1998 ▶). For the isostructural nickel analog, see: Zhou *et al.* (2006 ▶).
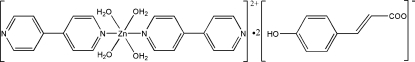


## Experimental

### 

#### Crystal data

[Zn(C_10_H_8_N_2_)_2_(H_2_O)_4_](C_9_H_7_O_3_)_2_
*M* *_r_* = 776.09
Triclinic, 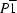

*a* = 7.0884 (4) Å
*b* = 7.3966 (4) Å
*c* = 17.2518 (10) Å
α = 86.972 (3)°
β = 83.872 (3)°
γ = 81.937 (3)°
*V* = 889.80 (9) Å^3^
*Z* = 1
Mo *K*α radiation
μ = 0.76 mm^−1^
*T* = 296 K
0.38 × 0.19 × 0.10 mm

#### Data collection

Bruker APEXII area-detector diffractometer
Absorption correction: multi-scan (*SADABS*; Sheldrick, 1996 ▶) *T*_min_ = 0.84, *T*_max_ = 0.93
12766 measured reflections
4058 independent reflections
3831 reflections with *I* > 2σ(*I*)
*R*_int_ = 0.020

#### Refinement

*R*[*F* ^2^ > 2σ(*F* ^2^)] = 0.029
*wR*(*F* ^2^) = 0.080
*S* = 1.03
4058 reflections
256 parameters
7 restraints
H atoms treated by a mixture of independent and constrained refinement
Δρ_max_ = 0.25 e Å^−3^
Δρ_min_ = −0.33 e Å^−3^

### 

Data collection: *APEX2* (Bruker, 2006 ▶); cell refinement: *SAINT* (Bruker, 2006 ▶); data reduction: *SAINT*; program(s) used to solve structure: *SHELXS97* (Sheldrick, 2008 ▶); program(s) used to refine structure: *SHELXL97* (Sheldrick, 2008 ▶); molecular graphics: *SHELXTL* (Sheldrick, 2008 ▶); software used to prepare material for publication: *SHELXTL*.

## Supplementary Material

Crystal structure: contains datablocks I, global. DOI: 10.1107/S1600536809023204/at2810sup1.cif

Structure factors: contains datablocks I. DOI: 10.1107/S1600536809023204/at2810Isup2.hkl

Additional supplementary materials:  crystallographic information; 3D view; checkCIF report

## Figures and Tables

**Table 1:** Hydrogen-bond geometry (Å, °)

*D*—H⋯*A*	*D*—H	H⋯*A*	*D*⋯*A*	*D*—H⋯*A*
O1*W*—H1*WA*⋯O3	0.818 (15)	1.948 (16)	2.7549 (15)	169 (2)
O1*W*—H1*WB*⋯O3^i^	0.835 (14)	1.875 (15)	2.7069 (14)	174 (2)
O1—H1⋯N1^ii^	0.824 (17)	1.97 (2)	2.714 (2)	150 (3)
O2*W*—H2*WA*⋯O2^iii^	0.834 (14)	1.869 (15)	2.6833 (14)	165.0 (19)
O2*W*—H2*WB*⋯O2^iv^	0.823 (14)	1.919 (15)	2.7307 (15)	168.3 (19)

Symmetry codes: (i) 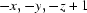; (ii) 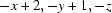; (iii) 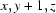; (iv) 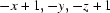.

**Fig. 1.**
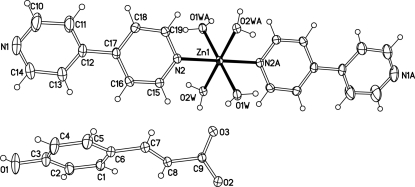
The cation and anion in (I), showing the atom-numbering scheme. Displacement ellipsoids are shown at the 30% probability level. [Symmetry code: (A) - x,1 - y,1 - z.]

**Fig. 2.**
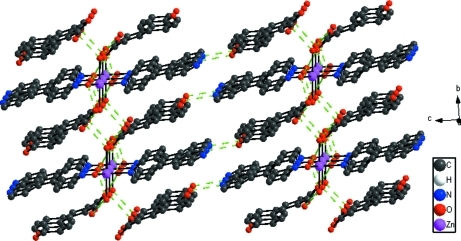
The crystal packing of (I). The dashed lines indicate hydrogen-bonding interactions. H atoms have been omitted for clarity.

## supplementary crystallographic information

### Comment

The main strategy widely used in design and synthesis of novel coordination architectures is the building-block approach (Yaghi *et al.*, 1998; Han *et al.*, 2005; Wen *et al.*, 2005). 4-Hydroxylcinnamic acid (H_2_L) is considered as suitable multidentate ligand is based on the following considerations: (*a*) It has multiple coordination sites, carboxylate group and phenolic hydroxyl group, that may generate structures of higher dimensions. (*b*) Hydroxyl group can also introduce hydrogen bond in the framework construction. Here, we combined H_2_L and auxiliary ligand 4,4-bipy as a mixed ligand system to react metal ions. A new Zn(II) complex, [Zn(4,4'-bipy)_2_(H_2_O)_4_].2HL, (I), was obtained unexpected. In this complex, HL ligand is non-coordinated and acts as a dissociative anion.

The X-ray diffraction study shows that the asymmetric unit of (I) is composed of half a Zn atom, one 4,4'-bipy ligand, two coordinated water molecules and one HL ligand. As shown in Fig.1, the Zn^II^ center is six-coordinated by four water molecules and two N atoms of 4,4'-bipy, and displays a slightly distorted [ZnO_4_N_2_] octahedral coordination geometry. Four water molecules form a relatively normal equatorial plane of the octahedron, and the Zn1 atom is located in this plane, while two N atoms occupy the axial positions, with an N—Zn—N angle of 180 °. The bond lengths of Zn—O_water_ are 2.0878 (10) and 2.0881 (10) Å, Zn—N is 2.1728 (12) Å, respectively.

There are extensive hydrogen-bonding interactions involving the HL oxygen atoms, coordinated water molecules and uncoordinated 4,4'-bipy N atoms. A three-dimensional network is formed by these hydrogen-bonding interactions, as shown in Fig. 2. Complex (I) is isostructural with its nickel analog (Zhou *et al.*, 2006).

### Experimental

A mixture of 4-hydroxylcinnamic acid (0.1642 g, 1 mmol), ZnSO_4_.7H_2_O (0.1438 g, 0.5 mmol), Na_2_CO_3 _(0.053 g, 0.5 mmol) and H_2_O (15 mL) was sealed in a 25 ml stainless-steel reactor with a Telflon liner and was heated at 433 K for 3 d. On completion of the reaction, the reactor was cooled slowly to room temperature and the mixture was filtered, giving colourless single crystals suitable for X-ray analysis in yield 30% (based on Zn).

### Refinement

The Carbon-bound H-atoms were positioned geometrically and included in the refinement using a riding model [C—H = 0.93 Å *U*_iso_(H) = 1.2*U*_eq_(C)]. The water and hydroxyl H atoms were located from different maps, and refined with O—H and H—H distances retrained to 0.85 (2) Å and 1.35 (2) Å, and *U*_iso_(H) values of 1.5*U*_eq_(O_water, hydroxyl_).

### Crystal data

**Table:**

[Zn(C_10_H_8_N_2_)_2_(H_2_O)_4_](C_9_H_7_O_3_)_2_	*Z* = 1
*M**_r_* = 776.09	*F*(000) = 404
Triclinic, *P*1	*D*_x_ = 1.448 Mg m^−^^3^
Hall symbol: -P 1	Mo *K*α radiation, λ = 0.71073 Å
*a* = 7.0884 (4) Å	Cell parameters from 7604 reflections
*b* = 7.3966 (4) Å	θ = 2.4–27.6°
*c* = 17.2518 (10) Å	µ = 0.76 mm^−^^1^
α = 86.972 (3)°	*T* = 296 K
β = 83.872 (3)°	Block, colourless
γ = 81.937 (3)°	0.38 × 0.19 × 0.10 mm
*V* = 889.80 (9) Å^3^	

### Data collection

**Table:**

Bruker APEXII area-detector diffractometer	4058 independent reflections
Radiation source: fine-focus sealed tube	3831 reflections with *I* > 2σ(*I*)
graphite	*R*_int_ = 0.020
φ and ω scans	θ_max_ = 27.6°, θ_min_ = 2.4°
Absorption correction: multi-scan (*SADABS*; Sheldrick, 1996)	*h* = −8→9
*T*_min_ = 0.84, *T*_max_ = 0.93	*k* = −9→9
12766 measured reflections	*l* = −22→22

### Refinement

**Table:**

Refinement on *F*^2^	Primary atom site location: structure-invariant direct methods
Least-squares matrix: full	Secondary atom site location: difference Fourier map
*R*[*F*^2^ > 2σ(*F*^2^)] = 0.029	Hydrogen site location: inferred from neighbouring sites
*wR*(*F*^2^) = 0.080	H atoms treated by a mixture of independent and constrained refinement
*S* = 1.03	*w* = 1/[σ^2^(*F*_o_^2^) + (0.0476*P*)^2^ + 0.2285*P*] where *P* = (*F*_o_^2^ + 2*F*_c_^2^)/3
4058 reflections	(Δ/σ)_max_ < 0.001
256 parameters	Δρ_max_ = 0.25 e Å^−^^3^
7 restraints	Δρ_min_ = −0.32 e Å^−^^3^

### Special details

**Table:**

Geometry. All e.s.d.'s (except the e.s.d. in the dihedral angle between two l.s. planes) are estimated using the full covariance matrix. The cell e.s.d.'s are taken into account individually in the estimation of e.s.d.'s in distances, angles and torsion angles; correlations between e.s.d.'s in cell parameters are only used when they are defined by crystal symmetry. An approximate (isotropic) treatment of cell e.s.d.'s is used for estimating e.s.d.'s involving l.s. planes.
Refinement. Refinement of *F*^2^ against ALL reflections. The weighted *R*-factor *wR* and goodness of fit *S* are based on *F*^2^, conventional *R*-factors *R* are based on *F*, with *F* set to zero for negative *F*^2^. The threshold expression of *F*^2^ > σ(*F*^2^) is used only for calculating *R*-factors(gt) *etc*. and is not relevant to the choice of reflections for refinement. *R*-factors based on *F*^2^ are statistically about twice as large as those based on *F*, and *R*- factors based on ALL data will be even larger.

### Fractional atomic coordinates and isotropic or equivalent isotropic displacement parameters (Å^2^)

**Table:**

	*x*	*y*	*z*	*U*_iso_*/*U*_eq_	
Zn1	0.0000	0.5000	0.5000	0.02490 (8)	
N1	0.7033 (3)	0.7037 (3)	0.02925 (11)	0.0716 (6)	
N2	0.15006 (17)	0.51181 (16)	0.38363 (7)	0.0286 (2)	
O1W	0.04162 (15)	0.21489 (14)	0.50894 (7)	0.0353 (2)	
H1WA	0.103 (3)	0.148 (3)	0.4760 (11)	0.053*	
H1WB	−0.050 (2)	0.165 (3)	0.5304 (11)	0.053*	
O1	1.2210 (3)	0.2418 (3)	0.12712 (9)	0.0791 (5)	
H1	1.211 (4)	0.235 (4)	0.0803 (11)	0.095*	
O2W	0.25236 (14)	0.50834 (15)	0.55126 (6)	0.0321 (2)	
H2WA	0.302 (3)	0.600 (2)	0.5343 (12)	0.048*	
H2WB	0.336 (3)	0.419 (2)	0.5482 (12)	0.048*	
O2	0.44862 (15)	−0.23700 (15)	0.47767 (7)	0.0375 (2)	
O3	0.24175 (14)	−0.04752 (14)	0.41292 (6)	0.0343 (2)	
C1	0.8995 (2)	0.1118 (2)	0.28991 (9)	0.0364 (3)	
H1A	0.8960	0.1062	0.3440	0.044*	
C2	1.0548 (2)	0.1715 (2)	0.24627 (10)	0.0418 (4)	
H2A	1.1535	0.2067	0.2709	0.050*	
C3	1.0634 (3)	0.1792 (3)	0.16569 (10)	0.0469 (4)	
C4	0.9163 (3)	0.1275 (3)	0.13005 (10)	0.0563 (5)	
H4A	0.9223	0.1306	0.0759	0.068*	
C5	0.7585 (3)	0.0705 (3)	0.17450 (10)	0.0472 (4)	
H5A	0.6582	0.0391	0.1496	0.057*	
C6	0.7480 (2)	0.0596 (2)	0.25546 (9)	0.0319 (3)	
C7	0.5805 (2)	−0.0009 (2)	0.30247 (9)	0.0329 (3)	
H7A	0.4696	−0.0010	0.2781	0.039*	
C8	0.5768 (2)	−0.0550 (2)	0.37683 (9)	0.0320 (3)	
H8A	0.6881	−0.0547	0.4009	0.038*	
C9	0.40854 (19)	−0.11645 (18)	0.42536 (8)	0.0274 (3)	
C10	0.5155 (4)	0.7408 (5)	0.03681 (14)	0.0983 (11)	
H10A	0.4563	0.7952	−0.0058	0.118*	
C11	0.4004 (3)	0.7038 (5)	0.10411 (13)	0.0830 (9)	
H11A	0.2681	0.7326	0.1056	0.100*	
C12	0.4808 (2)	0.6252 (2)	0.16828 (9)	0.0402 (4)	
C13	0.6782 (3)	0.5875 (3)	0.16090 (13)	0.0649 (6)	
H13A	0.7417	0.5348	0.2027	0.078*	
C14	0.7814 (3)	0.6288 (4)	0.09087 (15)	0.0740 (7)	
H14A	0.9141	0.6018	0.0874	0.089*	
C15	0.3318 (2)	0.4346 (2)	0.36835 (9)	0.0320 (3)	
H15A	0.3863	0.3548	0.4058	0.038*	
C16	0.4417 (2)	0.4678 (2)	0.29975 (9)	0.0358 (3)	
H16A	0.5680	0.4122	0.2920	0.043*	
C17	0.3643 (2)	0.5841 (2)	0.24213 (9)	0.0326 (3)	
C18	0.1735 (2)	0.6606 (2)	0.25723 (9)	0.0384 (3)	
H18A	0.1142	0.7375	0.2200	0.046*	
C19	0.0736 (2)	0.6215 (2)	0.32754 (9)	0.0364 (3)	
H19A	−0.0535	0.6740	0.3366	0.044*	

### Atomic displacement parameters (Å^2^)

**Table:**

	*U*^11^	*U*^22^	*U*^33^	*U*^12^	*U*^13^	*U*^23^
Zn1	0.02173 (12)	0.02703 (12)	0.02486 (13)	−0.00473 (8)	0.00350 (8)	0.00093 (8)
N1	0.0743 (13)	0.0967 (15)	0.0436 (10)	−0.0347 (11)	0.0228 (9)	0.0019 (10)
N2	0.0265 (6)	0.0313 (6)	0.0265 (6)	−0.0037 (4)	0.0028 (4)	−0.0002 (5)
O1W	0.0317 (5)	0.0268 (5)	0.0448 (6)	−0.0058 (4)	0.0115 (5)	−0.0023 (4)
O1	0.0665 (10)	0.1314 (16)	0.0453 (8)	−0.0548 (10)	0.0178 (7)	0.0054 (9)
O2W	0.0242 (5)	0.0359 (5)	0.0356 (6)	−0.0068 (4)	0.0003 (4)	0.0042 (4)
O2	0.0299 (5)	0.0385 (6)	0.0417 (6)	−0.0061 (4)	0.0016 (4)	0.0127 (5)
O3	0.0243 (5)	0.0373 (5)	0.0387 (6)	−0.0017 (4)	0.0041 (4)	0.0032 (4)
C1	0.0344 (8)	0.0457 (8)	0.0285 (7)	−0.0089 (6)	0.0010 (6)	0.0044 (6)
C2	0.0339 (8)	0.0531 (10)	0.0391 (9)	−0.0137 (7)	0.0016 (7)	0.0024 (7)
C3	0.0435 (9)	0.0587 (11)	0.0373 (9)	−0.0166 (8)	0.0113 (7)	0.0042 (8)
C4	0.0643 (12)	0.0818 (14)	0.0253 (8)	−0.0288 (11)	0.0065 (8)	0.0044 (8)
C5	0.0474 (10)	0.0654 (11)	0.0323 (8)	−0.0232 (8)	−0.0028 (7)	0.0032 (8)
C6	0.0305 (7)	0.0342 (7)	0.0294 (7)	−0.0051 (6)	0.0034 (6)	0.0040 (6)
C7	0.0274 (7)	0.0353 (7)	0.0353 (8)	−0.0061 (6)	0.0009 (6)	0.0026 (6)
C8	0.0234 (6)	0.0355 (7)	0.0359 (8)	−0.0055 (5)	0.0020 (6)	0.0047 (6)
C9	0.0255 (6)	0.0265 (6)	0.0291 (7)	−0.0044 (5)	0.0038 (5)	−0.0013 (5)
C10	0.0731 (17)	0.179 (3)	0.0418 (12)	−0.0355 (19)	0.0030 (11)	0.0382 (17)
C11	0.0505 (12)	0.153 (3)	0.0425 (12)	−0.0225 (14)	0.0033 (9)	0.0329 (14)
C12	0.0440 (9)	0.0449 (9)	0.0303 (8)	−0.0132 (7)	0.0112 (7)	−0.0007 (6)
C13	0.0474 (11)	0.0872 (16)	0.0521 (12)	−0.0041 (10)	0.0168 (9)	0.0156 (11)
C14	0.0547 (12)	0.0988 (18)	0.0613 (14)	−0.0142 (12)	0.0271 (11)	0.0082 (13)
C15	0.0299 (7)	0.0346 (7)	0.0291 (7)	−0.0009 (6)	0.0016 (6)	0.0024 (6)
C16	0.0273 (7)	0.0430 (8)	0.0337 (8)	0.0004 (6)	0.0056 (6)	−0.0009 (6)
C17	0.0340 (7)	0.0351 (7)	0.0275 (7)	−0.0075 (6)	0.0068 (6)	−0.0015 (6)
C18	0.0377 (8)	0.0445 (8)	0.0289 (7)	0.0018 (6)	0.0020 (6)	0.0073 (6)
C19	0.0291 (7)	0.0449 (8)	0.0311 (8)	0.0029 (6)	0.0032 (6)	0.0035 (6)

### Geometric parameters (Å, °)

**Table:**

Zn1—O1W	2.0878 (10)	C4—H4A	0.9300
Zn1—O1W^i^	2.0878 (10)	C5—C6	1.389 (2)
Zn1—O2W	2.0881 (10)	C5—H5A	0.9300
Zn1—O2W^i^	2.0881 (10)	C6—C7	1.4740 (19)
Zn1—N2^i^	2.1728 (12)	C7—C8	1.322 (2)
Zn1—N2	2.1728 (12)	C7—H7A	0.9300
N1—C14	1.312 (3)	C8—C9	1.4931 (18)
N1—C10	1.314 (3)	C8—H8A	0.9300
N2—C15	1.3388 (18)	C10—C11	1.384 (3)
N2—C19	1.3396 (19)	C10—H10A	0.9300
O1W—H1WA	0.818 (15)	C11—C12	1.365 (3)
O1W—H1WB	0.835 (14)	C11—H11A	0.9300
O1—C3	1.364 (2)	C12—C13	1.381 (3)
O1—H1	0.824 (17)	C12—C17	1.484 (2)
O2W—H2WA	0.834 (14)	C13—C14	1.386 (3)
O2W—H2WB	0.823 (14)	C13—H13A	0.9300
O2—C9	1.2605 (17)	C14—H14A	0.9300
O3—C9	1.2559 (17)	C15—C16	1.376 (2)
C1—C2	1.377 (2)	C15—H15A	0.9300
C1—C6	1.390 (2)	C16—C17	1.387 (2)
C1—H1A	0.9300	C16—H16A	0.9300
C2—C3	1.383 (2)	C17—C18	1.394 (2)
C2—H2A	0.9300	C18—C19	1.376 (2)
C3—C4	1.373 (3)	C18—H18A	0.9300
C4—C5	1.390 (2)	C19—H19A	0.9300
			
O1W—Zn1—O1W^i^	180.0	C5—C6—C7	120.99 (14)
O1W—Zn1—O2W	90.44 (4)	C1—C6—C7	121.73 (14)
O1W^i^—Zn1—O2W	89.56 (4)	C8—C7—C6	124.58 (14)
O1W—Zn1—O2W^i^	89.56 (4)	C8—C7—H7A	117.7
O1W^i^—Zn1—O2W^i^	90.44 (4)	C6—C7—H7A	117.7
O2W—Zn1—O2W^i^	180.0	C7—C8—C9	125.33 (14)
O1W—Zn1—N2^i^	86.05 (4)	C7—C8—H8A	117.3
O1W^i^—Zn1—N2^i^	93.95 (4)	C9—C8—H8A	117.3
O2W—Zn1—N2^i^	88.47 (4)	O3—C9—O2	124.73 (12)
O2W^i^—Zn1—N2^i^	91.53 (4)	O3—C9—C8	120.02 (13)
O1W—Zn1—N2	93.95 (4)	O2—C9—C8	115.24 (12)
O1W^i^—Zn1—N2	86.05 (4)	N1—C10—C11	124.1 (2)
O2W—Zn1—N2	91.53 (4)	N1—C10—H10A	117.9
O2W^i^—Zn1—N2	88.47 (4)	C11—C10—H10A	117.9
N2^i^—Zn1—N2	180.0	C12—C11—C10	120.1 (2)
C14—N1—C10	116.03 (18)	C12—C11—H11A	120.0
C15—N2—C19	117.08 (12)	C10—C11—H11A	120.0
C15—N2—Zn1	121.81 (10)	C11—C12—C13	116.05 (17)
C19—N2—Zn1	120.28 (9)	C11—C12—C17	122.30 (16)
Zn1—O1W—H1WA	124.9 (15)	C13—C12—C17	121.65 (17)
Zn1—O1W—H1WB	116.7 (14)	C12—C13—C14	119.7 (2)
H1WA—O1W—H1WB	109.5 (18)	C12—C13—H13A	120.2
C3—O1—H1	106 (2)	C14—C13—H13A	120.2
Zn1—O2W—H2WA	110.0 (14)	N1—C14—C13	124.0 (2)
Zn1—O2W—H2WB	118.7 (14)	N1—C14—H14A	118.0
H2WA—O2W—H2WB	108.1 (17)	C13—C14—H14A	118.0
C2—C1—C6	121.95 (15)	N2—C15—C16	123.05 (14)
C2—C1—H1A	119.0	N2—C15—H15A	118.5
C6—C1—H1A	119.0	C16—C15—H15A	118.5
C1—C2—C3	119.85 (16)	C15—C16—C17	120.05 (13)
C1—C2—H2A	120.1	C15—C16—H16A	120.0
C3—C2—H2A	120.1	C17—C16—H16A	120.0
O1—C3—C4	124.55 (16)	C16—C17—C18	116.86 (13)
O1—C3—C2	115.97 (17)	C16—C17—C12	121.15 (14)
C4—C3—C2	119.48 (15)	C18—C17—C12	121.99 (14)
C3—C4—C5	120.35 (16)	C19—C18—C17	119.57 (14)
C3—C4—H4A	119.8	C19—C18—H18A	120.2
C5—C4—H4A	119.8	C17—C18—H18A	120.2
C6—C5—C4	121.10 (17)	N2—C19—C18	123.35 (14)
C6—C5—H5A	119.5	N2—C19—H19A	118.3
C4—C5—H5A	119.5	C18—C19—H19A	118.3
C5—C6—C1	117.26 (14)		
			
O1W—Zn1—N2—C15	−57.34 (12)	C14—N1—C10—C11	−0.7 (5)
O1W^i^—Zn1—N2—C15	122.66 (12)	N1—C10—C11—C12	0.5 (6)
O2W—Zn1—N2—C15	33.20 (12)	C10—C11—C12—C13	0.1 (4)
O2W^i^—Zn1—N2—C15	−146.80 (12)	C10—C11—C12—C17	179.5 (3)
O1W—Zn1—N2—C19	133.37 (12)	C11—C12—C13—C14	−0.4 (4)
O1W^i^—Zn1—N2—C19	−46.63 (12)	C17—C12—C13—C14	−179.8 (2)
O2W—Zn1—N2—C19	−136.08 (12)	C10—N1—C14—C13	0.4 (4)
O2W^i^—Zn1—N2—C19	43.92 (12)	C12—C13—C14—N1	0.2 (4)
C6—C1—C2—C3	−0.6 (3)	C19—N2—C15—C16	2.1 (2)
C1—C2—C3—O1	179.30 (18)	Zn1—N2—C15—C16	−167.54 (12)
C1—C2—C3—C4	0.2 (3)	N2—C15—C16—C17	−0.9 (2)
O1—C3—C4—C5	−178.0 (2)	C15—C16—C17—C18	−0.9 (2)
C2—C3—C4—C5	0.9 (3)	C15—C16—C17—C12	178.75 (15)
C3—C4—C5—C6	−1.8 (3)	C11—C12—C17—C16	164.8 (2)
C4—C5—C6—C1	1.4 (3)	C13—C12—C17—C16	−15.9 (3)
C4—C5—C6—C7	180.00 (17)	C11—C12—C17—C18	−15.6 (3)
C2—C1—C6—C5	−0.2 (2)	C13—C12—C17—C18	163.7 (2)
C2—C1—C6—C7	−178.80 (15)	C16—C17—C18—C19	1.3 (2)
C5—C6—C7—C8	163.73 (17)	C12—C17—C18—C19	−178.31 (16)
C1—C6—C7—C8	−17.7 (2)	C15—N2—C19—C18	−1.6 (2)
C6—C7—C8—C9	179.92 (14)	Zn1—N2—C19—C18	168.18 (13)
C7—C8—C9—O3	−32.7 (2)	C17—C18—C19—N2	−0.1 (3)
C7—C8—C9—O2	147.76 (16)		

Symmetry codes: (i) −*x*, −*y*+1, −*z*+1.

### Hydrogen-bond geometry (Å, °)

**Table:**

*D*—H···*A*	*D*—H	H···*A*	*D*···*A*	*D*—H···*A*
O1W—H1WA···O3	0.82 (2)	1.95 (2)	2.7549 (15)	169 (2)
O1W—H1WB···O3^ii^	0.84 (1)	1.88 (2)	2.7069 (14)	174 (2)
O1—H1···N1^iii^	0.82 (2)	1.97 (2)	2.714 (2)	150 (3)
O2W—H2WA···O2^iv^	0.83 (1)	1.87 (2)	2.6833 (14)	165 (2)
O2W—H2WB···O2^v^	0.82 (1)	1.92 (2)	2.7307 (15)	168 (2)

Symmetry codes: (ii) −*x*, −*y*, −*z*+1; (iii) −*x*+2, −*y*+1, −*z*; (iv) *x*, *y*+1, *z*; (v) −*x*+1, −*y*, −*z*+1.
